# Dietary *Ulva lactuca* and CAZyme supplementation improve serum biochemical profile and hepatic composition of weaned piglets

**DOI:** 10.1038/s41598-023-36008-4

**Published:** 2023-05-31

**Authors:** David M. Ribeiro, Paula A. Lopes, Rui M. A. Pinto, José M. Pestana, Mónica M. Costa, Cristina M. Alfaia, Miguel P. Mourato, André M. de Almeida, João P. B. Freire, José A. M. Prates

**Affiliations:** 1 LEAF - Linking Landscape, Environment, Agriculture and Food Research Center, Associated Laboratory TERRA, Instituto Superior de Agronomia, Universidade de Lisboa, Tapada da Ajuda, 1349-017 Lisboa, Portugal; 2 CIISA - Centre for Interdisciplinary Research in Animal Health, Faculdade de Medicina Veterinária, Universidade de Lisboa, 1300-477 Lisboa, Portugal; 3 Laboratório Associado para Ciência Animal e Veterinária (AL4AnimalS), Lisboa, Portugal; 4 iMED.UL, Faculdade de Farmácia, Universidade de Lisboa, Avenida Professor Gama Pinto, 1649-003 Lisboa, Portugal; 5 JCS, Laboratório de Análises Clínicas Dr. Joaquim Chaves, Avenida General Norton de MatosMiraflores, 1495-148 Algés, Portugal

**Keywords:** Zoology, Animal physiology

## Abstract

*Ulva lactuca* is a seaweed with antinutritional cell wall for monogastrics. Carbohydrate-Active enZymes (CAZymes) supplementation can potentially cause its disruption. This study evaluates four diets: Ctrl—control diet; UL—control + 7% *U. lactuca* (wild caught, powdered form); ULR—UL + 0.005% Rovabio^®^ Excel AP; ULU—UL + 0.01% ulvan lyase on piglets’ haematologic and serologic profiles, hepatic lipids and minerals. White blood cells and lymphocytes reached the highest values in piglets fed UL compared to control, and to control and ULR; respectively (*P* < 0.05). IgG levels were boosted by seaweed incorporation compared to control (*P* = 0.015). The glycaemic homeostasis was assured by the seaweed inclusion. Dietary seaweed decreased serum lipids (*P* < 0.001), with the exception of ULU, due to HDL-cholesterol increase (*P* < 0.001). Cortisol was decreased in ULR and ULU (*P* < 0.001). No systemic inflammation was observed (*P* > 0.05). While hepatic *n*-3 PUFA increased in piglets fed with seaweed diets due to increment of beneficial 22:5*n*-3 and 22:6*n*-3 fatty acids (*P* < 0.05), the opposite occurred for *n*-6 PUFA, PUFA/SFA and *n*-6/*n*-3 ratios (*P* < 0.05). Hepatic pigments were unchanged (*P* > 0.05). ULR reduced α-tocopherol levels (*P* = 0.036) and increased serum potassium levels (*P* < 0.001) compared to control. Seaweed contributed to overcome piglets’ weaning stress, with some benefits of including CAZyme supplementation.

## Introduction

The environmental impact of livestock production has become an important and controversial global issue^1^, whose effects are expected to worsen as both world population and animal product demand increase. High quality and locally produced feedstuffs can be alternatives to conventional ones, such as maize or soybean meal that are imported for instance into Europe from the Americas with high economic and environmental costs. Seaweeds are among such alternatives. They are interesting, albeit heterogeneous, organisms providing an abundant source of biomass^2^. However, they have a recalcitrant cell wall with a complex cross-linked matrix of insoluble polysaccharides that reduce the digestive availability of intracellular nutrients during monogastrics’ digestion, thus limiting dietary incorporation levels for highly productive animals^3^. The supplementation with Carbohydrate-Active enZymes (CAZymes) stands out as a possible approach to increase the digestive utilization of such feedstuffs at higher inclusion levels in the feed^4^. Seaweeds are capable of improving quality traits and nutritional value of meat as well as animal health^2^, as previously described for *Laminaria digitata*^5^ and similar to some microalgae^6,7^.

The green seaweed *Ulva lactuca* is considered a good source of nutrients^2^. Like other edible green seaweeds, *U. lactuca*'s protein concentration, although very variable and depending on culture and environmental conditions, can represent up to 32% of the algae's dry mass^8^. This makes it a putative, partial alternative to conventional protein sources, such as soybean meal. Moreover, it contains a high amount of carbohydrates, such as ulvan with several bioactive properties (i.e., antibacterial, immunostimulant, antioxidant and antihyperlipidemic)^9^. This macroalga is also rich in vitamin B12, which plays a strong role in maintaining nervous system homeostasis or blood formation. Regarding other micronutrients, it is high in sodium, potassium, magnesium, iodine, manganese, and nickel and contains vitamin A, vitamin B1, vitamin C, calcium, phosphorous, and numerous other trace elements^10,11^. The recalcitrant cell wall of this seaweed makes it a challenge to allow monogastric animals to digest it, blocking endogenous enzymes access to its intracellular nutrients. Therefore, our research team has recently published promising in vitro results with ulvan lyase from PL25 family^12^. In fact, the reported twofold increase of 18:1*c*9 in the alga biomass when combined with ulvan lyase treatment in vitro warrants further research, due to the benefits of this fatty acid to hinder cardiovascular disease^13^. Furthermore, this supplementation can allow using this seaweed in the diets of animals with immature digestive systems, particularly the weaned piglet.

Weaning is a key period in which piglets face significant challenges that will determine their subsequent health and growth. In fact, once they are removed from the sow, they are transitioned from mostly liquid to mostly solid, plant-based feeding programs, in addition of being mixed with other litters in a new environment^14^. This is normally accompanied by anorexia and enteric inflammation^15,16^, often culminating in *post*-weaning diarrhoea. Therefore, these young animals require high-quality and digestible feedstuffs. In addition, with the recent European Union restrictions in using zinc oxide to deal with *post*-weaning stress^17^, bioactive feed ingredients are an increasingly important variable in dealing with this issue. Considering this, *U. lactuca* has the potential to provide energy and nutrients, while promoting piglet’s immune status and health.

Our research team has previously reported the effects of dietary microalgae with or without CAZyme supplementation on blood metabolites and hepatic lipids of weaned piglets^18,19^ and growing-finishing pigs^20^. For *Chlorella vulgaris*, the most important finding was the hepatic deposition of *n*-3 PUFA, in particular eicosapentaenoic acid (EPA), and the subsequent beneficial reduction of *n*-6/*n*-3 ratio^20^. In turn, dietary *Arthrospira platensis* increased systemic lipids, namely total cholesterol and LDL-cholesterol in weaned piglets, while boosting the antioxidant potential^19^. In view of these findings, we hypothesized that similar or additional beneficial effects could be obtained with the seaweed *U. lactuca*. Therefore, the main goal of this study was to determine the effects of *U. lactuca* at 7% of dietary incorporation, combined or not with CAZyme supplementation, on haematologic and serologic profiles, and hepatic lipid and mineral compositions of recently weaned piglets, thus ascertaining the impact of this seaweed on piglet’s nutrition and health.

## Results

### Zootechnical performance

The diets used in this study did not influence zootechnical performance of piglets (*P* > 0.05), including faecal consistency (Supplementary material 1). The average final weight of all piglets was 14.9 kg.

### Haematologic and serologic profiles

The effect of dietary *U. lactuca*, with or without CAZyme supplementation, on blood cells and serum biochemical metabolites from piglets is shown in Table 1. White blood cells count (*P* = 0.004) was higher in piglets fed *U. lactuca* diet mostly due to lymphocyte (*P* = 0.010) increase, reaching 53%. The opposite occurred for the granulocytes percentage (*P* = 0.009), which reached a minimum of 41.7%. Monocytes, red blood cells and thrombocytes were not affected (*P* > 0.05) by the diets.

**Table 1:** Effect of diets on haematologic and serologic profiles of piglets.

	Ctrl	UL	ULR	ULU	SEM	*P* value
Haematology						
White blood cells (× 10^9^/L)	15.3^a^	19.4^b^	18.0^ab^	17.6^ab^	0.440	0.004
Granulocytes (%)	48.0^a^	41.7^b^	48.4^a^	44.2^ab^	0.898	0.009
Lymphocytes (%)	48.2^a^	53.3^b^	48.0^a^	52.8^ab^	0.802	0.010
Monocytes (%)	2.60	3.50	2.80	2.67	0.227	0.480
Red blood cells (× 10^12^/L)	6.26	6.80	6.92	6.60	0.103	0.137
Haemoglobin (g/dL)	11.8	12.4	11.7	12.0	0.159	0.416
Thrombocytes (× 10^9^/L)	406	447	387	412	19.3	0.765
Serum metabolites						
Glucose (mg/dL)	123	117	123	120	1.60	0.502
Insulin (µU/mL)	< 0.4	< 0.4	< 0.4	< 0.4	–	–
HOMA-IR^3^ (mmol/L × µU/mL)	0.121	0.124	0.121	0.119	0.001	0.618
Urea (mg/dL)	6.70^a^	8.40^a^	7.20^a^	11.2^b^	0.439	< 0.001
Creatinine (mg/dL)	0.703^ab^	0.634^a^	0.707^ab^	0.739^b^	0.014	0.049
Cholesterol (mg/dL)	66.0^a^	62.0^a^	54.0^b^	72.9^c^	1.33	< 0.001
LDL-cholesterol (mg/dL)	37.1^a^	33.5^a^	24.5^b^	34.4^a^	0.932	< 0.001
HDL-cholesterol (mg/dL)	27.8^a^	26.6^a^	25.9^a^	33.0^b^	0.535	< 0.001
VLDL-cholesterol^1^ (mg/dL)	6.88^a^	6.04^b^	5.82^b^	7.00^a^	0.133	< 0.001
Total lipids^2^ (mg/dL)	316^a^	304^b^	287^c^	331^d^	2.90	< 0.001
TAG (mg/dL)	34.4^a^	30.2^b^	29.1^b^	35.0^a^	0.665	< 0.001
Albumin (g/dL)	3.16	3.13	2.95	2.98	0.048	0.324
Total protein (g/dL)	4.47^a^	4.55^a^	4.12^b^	4.13^b^	0.043	< 0.001
Hepatic markers						
ALT (U/L)	30.6	26.8	33.6	29.8	1.17	0.237
AST (U/L)	36.9^ab^	35.4^a^	43.8^b^	34.5^a^	1.06	0.004
GGT (U/L)	21.3^ac^	31.2^b^	17.8^a^	27.6^c^	1.27	< 0.001
ALP (U/L)	335^a^	202^b^	245^bc^	274^c^	10.4	< 0.001
Immunoglobulins						
IgA (mg/dL)	< 4.0	< 4.0	< 4.0	< 4.0	–	–
IgG (mg/dL)	91.9^a^	132^b^	101^ab^	111^ab^	4.78	0.015
IgM (mg/dL)	26.6^ab^	25.0^a^	30.4^b^	29.5^ab^	0.695	0.015
Hormones and inflammation markers						
Cortisol (µg/dL)	2.27^a^	1.95^a^	1.01^b^	0.924^b^	0.142	< 0.001
IGF-1 (µg/L)	191^a^	152^bc^	133^c^	171^ab^	5.21	< 0.001
IL-10 (pg/mL)	14.7^a^	21.2^b^	24.4^b^	29.4^c^	1.03	< 0.001
C-reactive protein (mg/dL)	< 0.03	< 0.03	< 0.03	< 0.03	–	–
IL-6 (pg/mL)	< 1.5	< 1.5	< 1.5	< 1.5	–	–
ApoA1 (mg/dL)	< 3.0	< 3.0	< 3.0	< 3.0	–	–
Electrolytes						
Na^+^ (mEq/L)	144	143	144	142	0.328	0.460
K^+^ (mEq/L)	6.50^a^	6.78^a^	7.46^b^	6.60^a^	0.094	< 0.001
Cl^−^ (mEq/L)	98.7^a^	101^ab^	102^b^	98.9^a^	0	0.017
Redox markers						
TAC (µM)	113	97.1	100	102	2.18	0.053
GPx (U/L)	490	567	526	612	25.5	0.378

^a^VLDL-cholesterol = 1/5 (TAG).

^b^Total lipids = (total cholesterol) × 1.12 + (TAG) × 1.33 + 1.48.

^c^HOMA-IR, insulin resistance index = [fasting serum glucose] × [fasting serum insulin]/22.5.

Ctrl—control diet; UL—control + 7% *Ulva lactuca*; ULR—UL + 0.005% Rovabio^®^ Excel AP; ULU—UL + 0.01% ulvan lyase; TAG—triacylglycerols; HDL—high-density lipoprotein; LDL—low density lipoprotein; VLDL—very low density lipoprotein; ALT—alanine aminotransferase; ALP—alkaline phosphatase; AST- aspartate aminotransferase; GGT—gamma-glutamyltransferase; IGF-1—insulin growth factor 1; IL-10—interleukin 10; IL-6—interleukin 6; IgA—immunoglobulin A; IgG—immunoglobulin G; IgM—immunoglobulin M; ApoA1—apolipoprotein A1. SEM—standard error of the mean. Values with different superscripts are significantly different at *P* < 0.05.

No change was detected for glucose, or insulin resistance index (HOMA-IR) (P > 0.05). Urea (*P* < 0.001) and creatinine (*P* = 0.049) were significantly increased in ULU (11.2 and 0.739 mg/dL, respectively) piglets compared respectively to all treatments and with UL. The same pattern of variation was found for cholesterol (*P* < 0.001), HDL-cholesterol (*P* < 0.001), VLDL-cholesterol (*P* < 0.001), total lipids (*P* < 0.001) and triacylglycerols (*P* < 0.001), in which piglets fed the combination of *U. lactuca* with the recombinant lyase had the highest values, whereas piglets fed the combination of *U. lactuca* with commercial Rovabio^®^ showed the lowest values. Still regarding lipemia, LDL-cholesterol (*P* < 0.001) was reduced by at least 26.9% with the combination of *U. lactuca* and commercial Rovabio^®^ by comparison to the other groups. Total protein was decreased by both feed enzymes relative to the other dietary groups (*P* < 0.001). Concerning hepatic markers, the highest values of alkaline phosphatase (ALP, *P* < 0.001) were seen in the control group (335 U/L) by comparison to the remaining diets. In turn, aspartate aminotransferase (AST, *P* = 0.004) had the highest levels in piglets fed the combination of *U. lactuca* with commercial Rovabio^®^ (43.8 U/L), while gamma-glutamyltransferase (GGT, *P* < 0.001) had the lowest levels in this same group (17.8 U/L). Regarding immunoglobulins, IgA was found below the minimum detection levels (< 4.0 mg/dL), IgM was lowest in *U. lactuca* without enzymes (25.0 mg/dL, *P* = 0.015), and IgG (*P* = 0.015) was decreased in the control group (91.9 mg/dL) by comparison to the *U. lactuca* dietary group (132 mg/dL). Cortisol (*P* < 0.001) was diminished by feed enzymes in relation to the other dietary treatments. *U. Lactuca* diets reduced IGF-1 (*P* < 0.001) concentrations by comparison to the control (191 µg/L). The interleukin-10 was higher in piglets fed *U. lactuca* combined with the recombinant ulvan lyase (29.4 pg/mL), intermediate in *U. lactuca* alone or combined with commercial Rovabio^®^ and lower in the control (*P* < 0.001). C-reactive protein (< 0.03 mg/dL), IL-6 (< 1.5 pg/mL) and ApoA1 (< 3.0 mg/dL) were found below the minimum detection levels. In terms of electrolytes, potassium (*P* < 0.001) and chloride (*P* = 0.017) were higher in piglets fed *U. lactuca* combined with commercial Rovabio^®^. Concerning redox status, piglets fed on the control diet tended to have higher concentrations of total antioxidant capacity (*P* = 0.053) when compared to piglets fed *U. lactuca*. In turn, glutathione peroxidase activity in piglets’ serum did not change between dietary treatments (P = 0.378).

### Hepatic total lipids, cholesterol and fatty acid profile

Cholesterol content and fatty acid profile in the liver are presented in Table 2. Contents of total lipid and total cholesterol were unchanged across dietary treatments (*P* > 0.05). Saturated fatty acids were highest in piglets fed the combination of *U. lactuca* with the recombinant lyase (63.8 g/100 g FA) when compared to the control (58.2 g/100 g FA), much at the expenses of 16:0 (*P* < 0.001) and 18:0 (P = 0.001) fatty acids. The reverse occurred for 15:0 (*P* < 0.001) and 17:0 (*P* < 0.001) fatty acids whilst 22:0 was higher in piglets fed the combination of *U. lactuca* with Rovabio^®^ (0.065 g/100 g FA), intermediate in the control (0.056 g/100 g FA), and *U. lactuca* combined with the recombinant ulvan lyase (0.059 g/100 g FA), and lower in piglets fed *U. lactuca* alone (0.034 g/100 g FA, *P* = 0.039). The sum of *cis* MUFA did not vary with diets (P > 0.05), although 17:1*c*9 content (*P* < 0.001) decreased in *U. lactuca* fed piglets, while 18:1*c*11 increased (*P* = 0.004). In turn, *n*-3 PUFA (*P* < 0.001) increased in piglets fed *U. lactuca* diets by up to 1.53-fold, the opposite occurred for *n*-6 PUFA (*P* = 0.012), total PUFA (*P* = 0.020), PUFA/SFA (*P* = 0.021) and *n*-6/*n*-3 (*P* < 0.001) ratio, which were mostly due to 18:2*n*-6 (*P* = 0.011), 20:2*n*-6 (*P* = 0.003) and 20:4*n*-6 (*P* = 0.021) variations. Finally, 22:*5n*-3 and 22:6*n*-3 fatty acids were increased by *U. lactuca* incorporation (0.330 and 0.662 g/100 g FA, respectively) compared to control (0.147 and 0.496 g/100 g FA, respectively).

**Table 2:** Effect of diets on the hepatic fatty acid profile of piglets.

	Ctrl	UL	ULR	ULU	SEM	*P*-value
Total lipids (g/100 g)	1.52	1.65	1.60	1.59	0.065	0.605
Total cholesterol (mg/100 g)	2.30	2.24	2.14	2.22	0.062	0.309
Fatty acid composition (g/100 g fatty acids)						
12:0	0.104	0.083	0.103	0.108	0.010	0.270
14:0	0.527	0.390	0.525	0.468	0.044	0.107
15:0	1.34^a^	0.570^b^	0.610^b^	0.603^b^	0.077	< 0.001
16:0	19.5^a^	22.0^ab^	23.7^b^	23.7^b^	0.680	< 0.001
16:1*c*7	0.345	0.331	0.371	0.364	0.023	0.615
16:1*c*9	0.951	0.999	1.15	0.953	0.083	0.319
17:0	8.13^a^	4.31^b^	3.70^b^	3.83^b^	0.394	< .001
17:1*c*9	1.67^a^	0.838^b^	0.742^b^	0.692^b^	0.128	< .001
18:0	28.5^a^	33.9^b^	34.3^b^	34.9^b^	1.18	0.001
18:1*c*9	13.9	14.7	14.8	14.6	0.476	0.555
18:1*c*11	2.06^a^	2.33^ab^	2.52^b^	2.43^b^	0.086	0.004
18:2*n*-6	11.2^a^	9.57^ab^	8.10^b^	8.40^b^	0.683	0.011
18:3*n*-6	0.086	0.079	0.076	0.068	0.008	0.432
18:2*t*9*t*12	0.108	0.101	0.091	0.099	0.011	0.747
18:3*n*-3	0.025	0.043	0.037	0.032	0.007	0.332
18:4*n*-3	0.041	0.057	0.050	0.050	0.012	0.841
20:0	0.118	0.099	0.119	0.110	0.010	0.488
20:1*c*11	0.197	0.168	0.180	0.167	0.010	0.115
20:2*n*-6	0.502^a^	0.326^b^	0.337^b^	0.307^b^	0.038	0.003
20:3*n*-6	0.369	0.295	0.255	0.221	0.046	0.141
20:4*n*-6	5.72^a^	4.83^ab^	3.70^ab^	3.50^b^	0.540	0.021
20:5*n*-3	0.126	0.189	0.205	0.190	0.024	0.108
22:0	0.056^ab^	0.034^a^	0.065^b^	0.059^ab^	0.008	0.039
22:1*n*-9	0.131	0.123	0.153	0.159	0.014	0.201
22:5*n*-3	0.147^a^	0.330^b^	0.249^bc^	0.195^ac^	0.026	0.000
22:6*n*-3	0.496^a^	0.662^b^	0.683^b^	0.693^b^	0.028	< .001
Other	3.69^a^	2.75^b^	3.19^ab^	3.05^ab^	0.189	0.010
Fatty acid partial sums						
SFA	58.2^a^	61.3^ab^	63.1^ab^	63.8^b^	1.31	0.021
*cis* MUFA	19.3	19.4	19.9	19.4	0.570	0.872
PUFA	18.8^a^	16.5^ab^	13.8^b^	13.8^b^	1.27	0.020
*n*-3 PUFA	0.837^a^	1.28^b^	1.22^b^	1.16^b^	0.036	< .001
*n*-6 PUFA	17.9^a^	15.1^ab^	12.5^b^	12.5^b^	1.26	0.012
Fatty acid ratios						
PUFA:SFA	0.335^a^	0.274^ab^	0.222^b^	0.217^b^	0.029	0.021
*n*-6:*n*-3	21.5^a^	11.9^b^	10.2^b^	10.8^b^	1.29	< .001

Ctrl—control diet; UL—control + 7% *Ulva lactuca*; ULR—UL + 0.005% Rovabio^®^ Excel AP; ULU—UL + 0.01% ulvan lyase; SEM—standard error of the mean. Values with different superscripts are significantly different at *P* < 0.05.

### Hepatic α-tocopherol and pigments

Contrarily to α-tocopherol, no differences were found for hepatic pigments (Table 3). α-Tocopherol was increased in the control group by 1.4-fold relative to piglets fed on *U. lactuca* combined with the commercial Rovabio^®^ (*P* = 0.036).

**Table 3:** Effect of diets on hepatic α-tocopherol and pigments of piglets.

	Ctrl	UL	ULR	ULU	SEM	*P*-value
α-Tocopherol (µg/100 g liver)	180^b^	141^a,b^	127^a^	141^a,b^	0.127	0.036
Pigments (µg/100 g liver)						
Chlorophyll-a	15.6	14.7	20.9	17.3	8.46	0.945
Chlorophyll-b	34.1	26.2	33.0	30.2	11.0	0.962
Total carotenoids	86.4	96.5	102	96.4	8.20	0.552

Ctrl—control diet; UL—control + 7% *Ulva lactuca*; ULR—UL + 0.005% Rovabio^®^ Excel AP; ULU—UL + 0.01% ulvan lyase. SEM—standard error of the mean. Values with different superscripts are significantly different at *P* < 0.05.

### Hepatic mineral profile

Micro- and macromineral profiles are presented in Table 4. No major variations were observed for mineral contents in the liver, except for calcium and sulphur. Calcium was increased in piglets fed the combination of *U. lactuca* with ulvan lyase (20.8 mg/100 g liver) relative to piglets fed *U. lactuca* alone (18.0 mg/100 g liver) *P* = 0.023). In turn, sulphur content was lower in piglets fed the control diet (162 mg/100 g liver) when compared to piglets fed *U. lactuca*, alone (184 mg/100 g liver) or combined with the commercial Rovabio^®^ (176 mg/100 g liver, *P* = 0.001).

**Table 4:** Effect of diets on the hepatic mineral profile (mg/100 g of liver) of piglets.

	Ctrl	UL	ULR	ULU	SEM	*P*-value
Macrominerals (mg/100 g of liver)						
Calcium (Ca)	18.3^ab^	18.0^a^	18.6^ab^	20.8^b^	0.688	0.023
Potassium (K)	334	342	341	343	4.31	0.491
Magnesium (Mg)	20.0	19.7	20.0	21.2	0.500	0.151
Sodium (Na)	78.9	75.9	73.5	76.1	1.76	0.214
Phosphorous (P)	346	363	341	330	9.05	0.097
Sulphur (S)	162^a^	184^b^	176^b^	175^ab^	3.44	0.001
Partial sum	959	1003	969	966	13.8	0.128
Microminerals (mg/100 g of liver)						
Copper (Cu)	2.62	2.47	1.88	1.70	0.297	0.098
Iron (Fe)	7.06	7.98	7.82	8.54	0.803	0.633
Manganese (Mn)	0.296	0.302	0.270	0.274	0.017	0.441
Zinc (Zn)	6.13	7.36	6.77	6.39	0.609	0.516
Partial sum	16.1	18.1	16.7	16.9	1.15	0.663
Total minerals	976	1021	986	983	14.1	0.117

Ctrl—control diet; UL—control + 7% *Ulva lactuca*; ULR—UL + 0.005% Rovabio^®^ Excel AP; ULU—UL + 0.01% ulvan lyase. SEM—standard error of the mean. Values with different superscripts are significantly different at *P* < 0.05.

### Principal component analysis (PCA)

**Figure 1:**
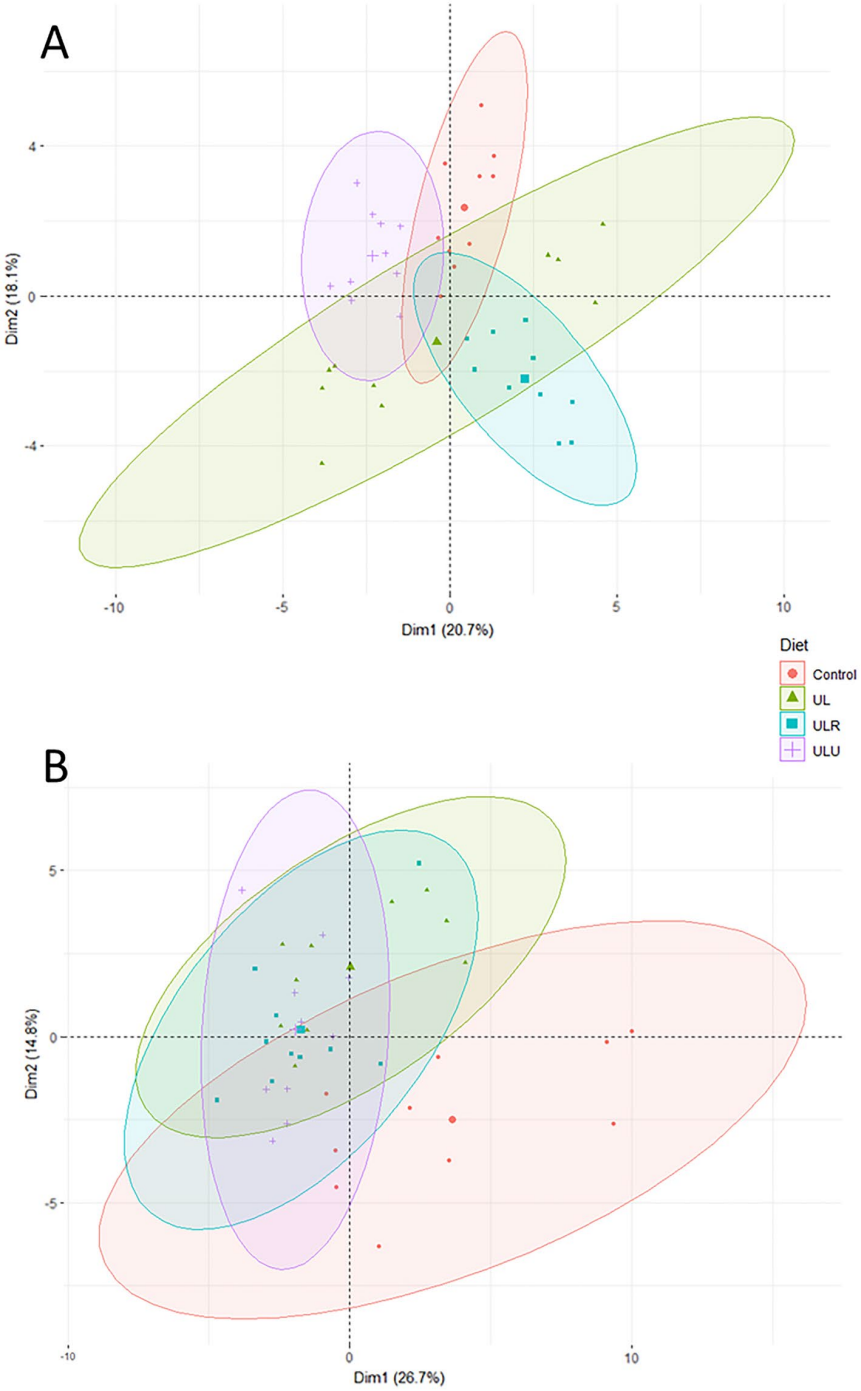
Principal component analysis (PCA) of serum profile (**A**) and liver (**B**) from piglets fed Ctrl—control diet; UL—control + 7% *Ulva lactuca*; ULR—UL + 0.005% Rovabio^®^ Excel AP; ULU—UL + 0.01% ulvan lyase.

Two principal component analyses, carried out with serum and liver metabolites, are depicted in Fig. 1 (panels A and B, respectively). The plot loadings are presented in supplementary material 2. Regarding serum, there was no clear clustering of any of the experimental groups. Nevertheless, there was a high heterogeneity of UL when compared to the other groups, particularly due to variables such as IgG and creatinine. The PCA carried out for liver metabolites revealed an overlap of all groups, but the controls tended to cluster separately, despite the high heterogeneity. Indeed, fatty acid variables such as 17:0, 15:0 and total *n*-6 PUFA explain such separation, which is in line with our previously mentioned statistical analysis, in which they were the highest in the control group.

## Discussion

To our knowledge, the use of *U. lactuca* seaweed as a feed ingredient, with 7% of dietary incorporation in weaned piglet’s diets has not been previously reported. Indeed, some studies have used *Ulva* sp. extracts to improve the immune status of weaner^21^ and weaned^22^ piglets but did not use the whole biomass. We initially hypothesized that feeding the seaweed at ingredient level (> 3%) without enzymatic supplementation might lead to antinutritional effects, causing lower availability (and consequent accumulation) of micronutrients, such as fatty acids. The objective of this study was to use it as a partial protein source for weaned piglets, taking advantage of its several bioactive properties in the process. The enzymatic supplementation aimed at maximizing its potential as a nutritional source. We therefore measured the piglet’s response in both blood and liver metabolites, thus focusing on systemic and central metabolism.

A moderate effect of experimental diets on blood cells was observed. Even if red blood cells and thrombocytes did not vary across dietary groups, lymphocytes increased as a consequence of dietary seaweed. On the contrary, the granulocytes count was reduced, and monocytes were kept unchanged in the experimental groups when compared to controls. A blood cell modulation of dietary seaweed has been previously reported by Shimazu et al*.*^23^ when applying 1% of dietary wakame (*Undaria pinnatifida*) which increased natural killer cells in pig’s blood. Our results seem to indicate that the seaweed promotes a positive modulation of the adaptative immune response, given that it increases lymphocytes by comparison to controls. However, the differences between the enzyme-supplemented groups were not significant, which suggested that the positive effect could be reversed. The reason for these variations is unclear.

Lipemia was influenced by dietary treatments. Interestingly, a consistent pattern of reduction was observed for total cholesterol, VLDL-cholesterol, total lipids and triacylglycerols levels when *U. lactuca* was fed to piglets, alone or combined with the commercial Rovabio^®^, but not with the recombinant ulvan lyase, by comparison to the control diet. In addition, LDL-cholesterol decreased across all *U. lactuca* diets. Even if total cholesterol marginally surpassed the reference values (36–54 mg/dL) for pigs^24^, such hypocholesterolaemic effect is known to reduce the risk of cardiovascular morbidity and human mortality^25^. In some clinical studies, *U. lactuca* has been established as a source of antioxidants capable of decreasing serum total cholesterol, LDL cholesterol, and triacylglycerols levels^11^, all of which are significant risk factors for coronary diseases. The reversion of this effect by the recombinant enzyme could originate the degradation of the *U. lactuca* cell wall, making the lipid fraction more available for digestion. This would explain, for example, the increased circulating triacylglycerol levels in the ulvan lyase group. In addition, no such increase was recorded in the Rovabio^®^ supplemented group, putatively demonstrating the inability of the commercial enzyme mix to efficiently degrade the recalcitrant seaweed polysaccharides.

No changes were detected either for glucose or for insulin resistance index (HOMA-IR), suggesting the maintenance of glycaemic homeostasis. This happened despite reduced insulin-like growth factor (IGF-1) levels by *U. lactuca* inclusion*,* with control and ulvan lyase groups being similar. Insulin-like growth factor has the ability to decrease blood glucose levels, which does not agree with our data. Insulin is a well-known stimulator of lipogenesis^26^ that increases fatty acid synthesis in the liver with formation and storage of triacylglycerols^27^. The reason why glucose levels were maintained might be putatively related to nutrient unavailability of seaweed.

Concerning hepatic function markers, different effects were promoted by feed enzymes. While the recombinant ulvan lyase decreased AST, the commercial Rovabio^®^ and *U. lactuca* alone decreased GGT and ALP, respectively, compared to the other diets. Despite the variations observed for aminotransferase activities, it is worth noticing that the levels found are still within the reference figures for pigs, which are 31–58 U/L for ALT, 32–84 U/L for AST, 10–52 U/L for GGT and 118–395 U/L for ALP, respectively^24^. Therefore, these differences do not seem to have biological significance.

As long described, algae inclusion in feeds has immune-modulatory properties. This is what is expected to occur. For instance, their anti-inflammatory activity has been associated with a minor secretion of pro-inflammatory cytokines^28,29^. This fact is corroborated by the absence of effects on IL-6, and also by enhanced levels of IL-10, across *U. lactuca* diets. IL-10 is a pleiotropic cytokine known for its potent anti-inflammatory and immunosuppressive effects^30^ that maintain normal tissue homeostasis^31^, while IL-6 is a multifunctional cytokine that plays a central role in host defense due to its wide range of immune and hematopoietic activities and its potent ability to induce the acute phase response^32^. Polysaccharides in *U. lactuca* have also been demonstrated to have significant analgesic and anti-inflammatory characteristics and may well be related with such differences. These are particularly relevant for the weaned piglet, given that they endure severe enteral inflammation associated with increased exposure to pathogens and undernutrition^16,33^.

Accordingly, we found that *U. lactuca* by itself increased IgG immunoglobulins, which are known to be the first line of defence of the organism against infections^34^ alongside with IgM. This might have contributed for increased serum protein content. IgG and IgM antibodies act in a coordinated way in short- and long-run protection against infections^35^. Upon infection, the IgM level will rise for a short period of time and then will begin to drop as the IgG levels increase, protecting the organism in the long-run^35^. In accordance, white blood cells, as a whole and lymphocytes, in particular were also increased in piglets fed *U. lactuca*-based diets. Bussy et al.^21^ reported that a sulphated polysaccharide extract from *U. armoricana* in sow diets increased serum IgG of nursing sows and their piglets. The partial degradation of the cell wall by targeting ulvan might explain why the effects of enzyme supplementation of our study reversed IgG increases in the non-supplemented diets. Indeed, since ulvan is a major cell wall polysaccharide and has immunomodulating properties^36^, its degradation will theoretically prevent its effects. Altogether, these changes suggest an improved immune response during the critical post-weaning period of piglets promoted by the seaweed, which is at least attenuated by the enzymatic supplementation.

Some n-3 fatty acids, which are highly present in marine algae, have immunomodulatory properties^37,38^. Moreover, antioxidants, β-carotene and vitamin B12 are also available in seaweeds, and can also modulate the immune system, even in different animal models^39^. In this study, IL-6, C-reactive protein and ApoA1 were found below detection limits. IL-6, a pro-inflammatory cytokine, is inhibited by long-chain n-3 PUFA, through the signalling of NF_k_B transcription factor^25^. ApoA1 is a protein involved in lipid metabolism, which is upregulated by n-3 PUFA signalling of PPARα in the liver^25^. The de novo lipogenesis that is putatively occurring in the liver because of dietary seaweed (discussed below) might provide fatty acids, such as DHA to signal these pathways. The absence of changes in these biomarkers might indicate that feeding *U. lactuca* does not trigger these inflammatory pathways. This is additionally supported by the fact that the acute phase C-reactive protein was unaffected by dietary treatments. All things considered, these variations reflect a boost on cellular and humoral immune response stimulated by *U. lactuca* that contribute to guarantee piglets’ survival at the critical post-weaning period.

Cortisol is a steroid hormone that regulates a wide range of fundamental processes, including metabolism and immune response. It also has a very important role in stress response^40,41^. Stress is a phenomenon with multifactorial causes, which produces an organic response that generates adverse effects in pigs’ health and welfare, and general productive performance. Herein, both exogenous feed enzymes displayed a beneficial effect in piglets by promoting a reduction of this stress hormone^42^. This could be related with decreased metabolic stress caused by higher nutrients availability, but more data is necessary to confirm this hypothesis.

Renal failure disorders are usually associated with common electrolytes, such as sodium and potassium^43^. Serum potassium was increased in piglets fed *U. lactuca* combined with commercial Rovabio^®^, whereas the reverse was found with the recombinant ulvan lyase. Additionally, the kidney plays a fundamental role in keeping chloride balance in the organism. Renal chloride transport is coupled with sodium transport^44^, in accordance with the values observed for both parameters in this study. Although statistically significant, the variations found for potassium in serum are believed to be devoid of clinical physiological relevance, as the mean values obtained are very similar among dietary treatments together with a minimal standard error.

The tendency for reduced antioxidant capacity in piglets fed *U. lactuca* combined with commercial Rovabio^®^ compared to controls was supported partially by a reduction in hepatic α-tocopherol, the most potent fat-soluble antioxidant available in nature^45^. No further variations in pigments with antioxidant function, like chlorophylls and carotenoids, were found in the liver, suggesting that they might be metabolized in another, hitherto unknown, fashion.

The hepatic fatty acid profile was significantly improved by the experimental diets. The most striking evidence is that *U. lactuca* based diets significantly increased *n*-3 PUFA in the liver, which explains the decreased *n*-6/*n*-3 ratio found in this tissue mostly due to 18:2*n*-6 (LA), 20:2*n*-6, and 20:4*n*-6 (AA) variations. Under the same rationale, 22:5*n*-3 (DPA) and 22:6*n*-3 (DHA) fatty acids were increased by the seaweed inclusion. Major *n*-3 PUFA, such as 18:3*n*-3 (ALA) and 18:*4n*-3, were present in high levels in *U. lactuca* diets, with the latter fatty acid not being detected in the control. Therefore, dietary availability is most likely the major driver for such changes. Overall, the aforementioned variations contribute to improved nutritional composition of this edible tissue, in addition to its health promoting properties for the weaned piglet. Indeed, *n*-3 PUFA accumulation could contribute to a downregulation of PUFA oxidation pathways and improved hepatic oxidative status^46^, counterbalancing the lower hepatic α-tocopherol levels found in seaweed diets. Similarly to what has been reported by Coelho et al*.*^20^ using 5% of dietary *Chlorella vulgaris* in growing-finishing pigs, increased hepatic *n*-3 PUFA contents could be at least partially explained due to increased intake of the major precursor of PUFA elongation and desaturation, the 18:*3n*-3 (ALA) fatty acid. This is additionally supported by the fact that we did not detect these long-chain PUFA in the diets but found them in the liver. Nevertheless, lowering the n-6/n-3 ratio ultimately improves animal health and nutritional quality of this edible tissue. Indeed, lowering it has been associated with reduced hepatic inflammation and increased fatty acid oxidation in the liver, contributing for reduced hepatic lipid accumulation^47^. It also contributes for an increased intake of n-3 PUFA by the consumer with known cardiovascular-health promoting effects^48^. Unexpectedly, 18:1*c*9 monounsaturated fatty acid was similar across dietary groups when a twofold increase was observed with the recombinant ulvan lyase *in vitro*^12^. The reasons for such a lack of differences are not clear.

In general, seaweeds have high mineral content, depending on several factors including species, environmental conditions and harvest season^2,3,49^. In this study, minor variations promoted by *U. lactuca* seaweed were found for mineral content in the liver. Indeed, microminerals, in particular copper, iron, manganese and zinc, presented no changes across dietary treatments. Copper and manganese are particularly important cofactors for antioxidant enzymes, such as superoxide dismutase^50,51^ which contributes to overall antioxidant capacity. The lack of differences in these microminerals could be associated with similar antioxidant enzyme activity in the four groups. Indeed, the only variations observed were for a few macrominerals, namely calcium and sulphur. Calcium levels were higher in piglets fed *U. lactuca* combined with the recombinant ulvan lyase by comparison with piglets fed on *U. lactuca* alone, possibly reflecting increased intracellular calcium availability. In turn, sulphur was increased by *U. lactuca* diets reflecting its composition in seaweed diets.

Finally, our PCAs performed with serum and liver metabolites demonstrate that there is no clear clustering of the experimental groups. However, *U. lactuca* alone and control tended to be separated from the other groups in serum and liver, respectively, despite being more heterogeneous. Overall, this could demonstrate that feeding piglets with this high level of dietary *U. lactuca* has no major detrimental effect in systemic and central metabolism of piglets, regardless of feed enzymes supplementation.

## Conclusions

Herein, we reported for the first time the effect of 7% of dietary *U. lactuca* in combination with feed enzymes in the haematological profile, serum biochemical metabolites, and hepatic lipid compounds, pigments, and mineral composition in recently weaned piglets. *U. lactuca* inclusion promoted a boost of the immune system, through the increase of lymphocytes and IgG immunoglobulins. This fact is corroborated by the non-variation of IL-6, and by the enhanced levels of IL-10, a pleiotropic cytokine known for its potent anti-inflammatory and immunosuppressive effects in the three *U. lactuca* fed groups. In addition, a hypolipidemic effect affecting total cholesterol, VLDL-cholesterol, total lipids and triacylglycerols was observed for *U. lactuca* dietary inclusion, alone or in combination with commercial Rovabio^®^, but not for the seaweed combined with the recombinant ulvan lyase. In addition, *U. lactuca* decreased the hepatic *n*-6/*n*-3 ratio, which is a consequence of high deposition of beneficial *n*-3 fatty acids (as the case of DPA and DHA). Hepatic pigments remained unchanged across dietary groups, but α-tocopherol was slightly reduced by *U. lactuca* reaching statistical significance when combined with Rovabio^®^.

Taken together, our findings indicate that *U. lactuca* at high incorporation levels is safe for weaned piglets, as no kidney or liver toxicity was recorded, and promotes a strong activation of their immune system, mediated by the increase of lymphocytes and IgG immunoglobulins. Also, *U. lactuca* does not affect glycaemic homeostasis and induces a hypolipidemic effect, involving both cholesterol and triacylglycerol compounds. In addition, both exogenous enzymes seem to display a beneficial effect on piglets’ stress by promoting a reduction in cortisol concentrations. Further research using the swine model is needed to clarify the mechanisms associated with the immunostimulatory, anti-inflammatory and hypolipidemic effects promoted by *U. lactuca*, combined or not with feed enzymes. Moreover, studying possible negative impacts of heavy metals accumulated by the seaweed on animal metabolism would be important to fully ascertain its feed safety. An integrated proteomics and metabolomics approach could putatively be of high relevance for the elucidation of such mechanisms.

## Materials and methods

### Experimental animal trial

The Animal Experimentation Ethics Commission of the Higher Institute of Agronomy of the University of Lisbon (Portugal) and the Portuguese National Veterinary Authority (Process Number 0421/000/000/2020) approved the experimental protocol. All methods were performed in accordance with relevant guidelines and regulations, including the European Union legislation (2010/63/EU Directive) and ARRIVE guidelines 2.0 (https://arriveguidelines.org/arrive-guidelines). The *U. lactuca* was bought from Aleor (Brittany, France), in a powder form (< 250 µm). It was low-temperature dried, milled and micronized before packaging. The experimental animal trial was carried out at the Animal Production Section of the Higher Institute of Agronomy, University of Lisbon, Portugal. Forty male piglets (Large White × Duroc), weaned at 28 days old and from different litters, were randomly allocated into four mash dietary treatments (*n* = 10): a control diet, UL (7% *Ulva lactuca*, replacing control), ULR (UL + 0.005% Rovabio^®^ Excel AP of Adisseo (Antony, France)) and ULU (UL + 0.01% ulvan lyase). No salt was added to seaweed diets. Control and *U. lactuca* diets had 18.0% and 18.3% crude protein on a dry matter (DM) basis and 5.6% and 5.5% of crude fat on a DM basis, respectively. The seaweed powder had 28.2% and 2.9% of crude protein and crude fat on a DM basis, respectively. The main ingredients of feeding treatments, fatty acid composition, pigments and minerals are presented in Table 5. Each piglet was individually housed in metabolic cages with free access to water. After 5 days of adaptation period to minimize stress and stabilize all metabolic conditions, the experimental animal trial began and lasted two weeks. During the trial, piglets were fed on a pair-feeding basis (50 g/kg of live weight) each day and were weighed at the beginning and end of each week. Faecal consistency was scored daily (0-normal faeces, 1-soft faeces, 2-diarrhoea, 3-severe diarrhoea). By the end of the experiment, all piglets were slaughtered according to standard commercial practices. Blood was collected into Sarstedt Z serum tubes (ref.: 32,329, Sarstedt, Nümbrecht, Germany) followed by centrifugation at 1500 g for 10 min at room temperature to separate serum which was frozen at − 80 °C, until further analyses. Hepatic samples were harvested from within the tissue, minced and frozen at − 80 °C.

**Table 5:** Main ingredients and composition of diets in fatty acids, pigments and minerals.

	*U. lactuca*	Ctrl	UL	ULR	ULU
Ingredients (g/kg)					
Wheat	–	437	407	406.95	406.9
Maize	–	150	140	140	140
Soybean meal 44	–	250	233.1	233.1	233.1
Sweet whey powder	–	100	93.4	93.4	93.4
Sunflower oil	–	30	28.5	28.5	28.5
*Ulva lactuca*	–	0	70	70	70
L-Lysine	–	5	4.7	4.7	4.7
DL-Methionine	–	1	0.9	0.9	0.9
L-Threonine	–	1	0.9	0.9	0.9
Calcium carbonate	–	5	4.7	4.7	4.7
Dicalcium phosphate	–	13	12.1	12.1	12.1
Sodium chloride	–	3	0	0	0
Vitamin-mineral premix^a^	–	5	4.7	4.7	4.7
Rovabio^®^ Excel AP	–	0	0	0.05	0
Ulvan lyase	–	0	0	0	0.1
Chemical composition (% of DM)^b^					
Crude protein	28.2	18.0	18.3	18.7	18.6
Ether extract	2.9	5.6	5.5	5.7	5.3
Amino acid content (g/kg)^c^					
Lysine	7.8	14.0	13.9	13.9	13.9
Methionine + Cysteine	5.8	7.0	6.9	6.9	6.9
Tryptophan	1.0	2.2	2.1	2.1	2.1
Threonine	2.8	7.0	7.0	7.0	7.0
Metabolizable energy (cal/g)^c^	–	3305	–	–	–
Fatty acid profile (% of total fatty acids)					
14:0	0.608	0.384	0.434	0.387	0.409
16:0	33.6	10.8	12.0	11.5	11.5
16:1*c*9	4.17	0.163	0.270	0.273	0.289
17:0	0.580	0.088	0.077	0.081	0.076
17:1*c*9	0.958	0.049	0.052	0.052	0.047
18:0	1.26	3.62	3.52	3.50	3.52
18:1*c*9	14.9	26.0	25.0	25.8	25.4
18:2*n-6*	6.14	55.2	52.7	52.8	53.1
18:3*n-3*	17.3	1.32	1.91	1.72	1.72
18:4*n*-3	12.8	0.000	0.619	0.599	0.578
20:0	0.344	0.320	0.336	0.329	0.323
22:0	1.54	0.638	0.637	0.645	0.627
Diterpene profile (µg/g)					
α-Tocopherol	79.3	52.4	46.8	47.3	51.0
β-Tocopherol	n.d	1.01	0.992	0.839	0.849
γ-Tocopherol + β-tocotrienol	n.d	2.39	2.38	1.93	1.94
δ-Tocopherol	n.d	0.522	0.508	0.457	0.487
γ-Tocotrienol	n.d	1.63	1.68	1.34	1.42
Pigments (µg/g)					
β-Carotene	170	0.441	14.5	16.7	15.2
Chlorophyll-*a*	2311	3.86	239	238	247
Chlorophyll-*b*	1666	5.80	166	167	170
Total Carotenoids	510	1.91	43	43	45
Macrominerals (mg/kg)					
Calcium	6202	14,661	14,966	15,017	14,011
Potassium	38,822	10,967	14,206	13,946	14,431
Magnesium	25,889	1456	4186	3980	4018
Sodium	52,133	4082	7486	7212	7618
Phosphorous	2786	8722	8876	9340	8638
Sulphur	49,265	2998	8906	8323	8720
Microminerals (mg/kg)					
Copper	3.73	256	240	258	227
Iron	537	265	258	294	253
Manganese	39.0	142	129	145	145
Zinc	8.96	257	261	256	281
Iodine	45.1	1.45	5.88	7.12	5.66
Bromine	694	11.7	77.3	86.3	81.8

Ctrl—control diet; UL—control + 7% *Ulva lactuca*; ULR—UL + 0.005% Rovabio^®^ Excel AP; ULU—UL + 0.01% ulvan lyase.

^a^Vitamin and trace mineral supplied *per* kilogram of diet—Vit. A: 25 000 IU; Vit. D3: 2000 IU; Vit. E: 20 IU; Vit C: 200 mg; Vit. B1: 1.5 mg; Vit. B2: 5 mg; Vit. B3: 30 mg, Vit. B5: 15 mg; Vit. B6: 2.5 mg; Vit. B9: 0.5 mg; Vit. B12: 0.03 mg; Vit. K3: 1 mg; Vit. H2: 80 mg; choline (chloride): 300 mg; I: 1 mg as potassium iodate; Mn: 50 mg as manganese (oxide); Fe: 120 mg as ferrous carbonate; Zn: 140 mg as zinc (oxide); Cu: 160 mg as copper sulphate; Se: 0.3 mg as sodium selenite; Co: 0.5 mg as cobalt carbonate.

^b^Determined as previously reported by Ribeiro et al.^5^.

^c^Calculated from reference values: *Ulva lactuca*^52^, remaining feedstuffs^53^.

### Blood biochemical parameters determination

Biochemical metabolites: triacylglycerols (TAG), total cholesterol, urea, total protein, albumin, LDL-cholesterol, HDL-cholesterol, glucose, insulin, hepatic markers, and creatinine concentrations were determined in serum using a Modular Hitachi Analytical System (Roche Diagnostics, Mannheim, Germany) and diagnostic kits (Roche Diagnostics, Meylan, France) following manufacturer’s instructions. VLDL-cholesterol and total lipids were determined using Covaci et al*.*^54^ and Friedewald et al*.*^55^ formulas. IgA, IgG and IgM were assessed by immunoturbidimetry. Total antioxidant capacity was determined by Quanti-Chrom Antioxidant Assay Kit (DTAC-100, Bioassay Systems, Hayward, CA, USA) and glutathione peroxidase activity (GPx) by EnzyChrom Glutathione Peroxidase Assay Kit (EGPx-100, Bioassay Systems). One unit of GPx is the amount of GPx that produces 1 μmol of glutathione disulphide (GSSG) per minute at pH = 7.6 and room temperature. White blood cells, red blood cells and thrombocytes counts were achieved through Sysmex XN-10 analysers (Sysmex Corporation, Kobe, Japan), as reported before^20^. Insulin growth factor-1 (IGF1), interleukin-6 (IL-6) and cortisol were determined by an electrochemiluminescence immunoassay kit (Roche Diagnostics, Meylan, France), as described^56^. Apolipoprotein A1 (ApoA1) and C-reactive protein were quantified by immunoturbidimetry (Roche Diagnostics, Meylan, France). IL-10 was determined with a DIASource (Louvain-la-Neuve, Belgium) immunoassay kit. The main electrolytes (Na^+^, K^+^ and Cl^-^) were determined by indirect potentiometry.

### Hepatic lipids determination

Freeze-dried hepatic samples were used for total lipid extraction, using the Folch et al.^57^ procedure with methanol and dichloromethane (1:2 v/v)^58^. Fatty acids were transesterified with NaOH in anhydrous methanol (0.5 M) followed by a solution of acetylchloride-methanol (1.25 M Sigma-Aldrich, St. Louis, Mo, USA) at 50 ºC, for 30 and 10 min, respectively^63^. Fatty acid methyl esters were determined by gas-chromatography (Hewlett-Packard, Palo Alto, CA, USA) with a flame-ionization detector and a Supelcowax^10^ capillary column (30 m × 0.20 mm i.d., 0.20 μm film thickness; Supelco Inc., Bellefonte, PA, USA)^59^. The nonadecanoic acid (19:0) was the internal standard, converting peak areas into weight percentages. The identification of fatty acids was done according to their retention times and expressed as g/100 g of total fatty acids.

### Hepatic pigments determinatio n

Chlorophylls a and b, and total carotenoids were determined using the Tolpeznikaite et al*.*^60^ protocol with minor modifications. In total, 2.5 g of fresh liver was weighted and 5 mL of 100% acetone added. Then, samples were homogenised with a T25 UltraTurrax homogenizer (IKA, Königswinter, Germany) and then centrifuged at 3000 rpm for 5 min at 4 ºC. The supernatant was separated and immediately analysed. These compounds were measured using UV–Vis spectrophotometry (ThermoScientific—Genesys 150, Waltham, MA, USA). The pigments content was determined using equations described by Dere et al*.*^61^.

### Hepatic minerals determination

Hepatic minerals were determined as described by Ribeiro et al*.*^62^. Briefly, 300 mg of freeze-dried liver was weighed into a digestion tube. HCl and HNO_3_ solutions were added to each tube, followed by an overnight incubation. Before digestion, H_2_O_2_ was added to each tube followed by 1 h of gradual increase to 95 ºC, and another hour at constant 95 °C. Afterwards, the subsequent solution was filtered and processed with inductively coupled plasma—optical emission spectrometry (ICP-OES).

### Statistical analysis

Data treatment was performed with Statistical Analysis Software, version 9.4 (SAS Institute, Cary, NC, USA) and analysed with the General Linear Model (GLM) procedure, using the piglet as the experimental unit. The effect of litter was also tested, but since it had no significant effect, it was removed from the model. Statistically significant differences were compared with the Tukey test of the PDIFF option. All data were presented as means with their standard errors (SEM). Statistical tests were significant at a probability level of 5%. Serum and hepatic metabolites were further processed for Principal Components Analysis with the RStudio software (version 2022.02.0 + 443), using the *FactoMineR* and *factoextra* packages, *fviz_pca_ind* function.

## Supplementary Information

Supplementary Table 1.

Supplementary Table 2.

## Data Availability

All data is contained in the article.
