# Supplementary material for: Dietary Ulva lactuca and CAZyme supplementation improve serum biochemical profile and hepatic composition of weaned piglets
Source: Sci Rep. 2023 May 31;13:8784. doi: 10.1038/s41598-023-36008-4 (PMC10232413; doi:10.1038/s41598-023-36008-4)
Supplement: Supplementary file 1 — Supplementary Table 1. [file 41598_2023_36008_MOESM1_ESM.docx]

**Supplementary material 1 – Growth performance of piglets**

Table 1 – Growth performance of piglets fed with control (wheat and maize-based), UL (7% *Ulva lactuca*), ULR (UL+0.005% Rovabio® Excel AP) and ULU (UL+0.01% ulvan lyase) diets.

|  | Control | UL | ULR | ULU | SEM | Litter | Diet |
| --- | --- | --- | --- | --- | --- | --- | --- |
| Number of piglets | 10 | 10 | 10 | 10 |  |  |  |
| Initial weight (kg) | 9.4 | 9.7 | 9.6 | 9.7 | 0.17 | 0.004 | 0.787 |
| Final weight (kg) | 14.7 | 15.0 | 14.9 | 14.9 | 0.30 | 0.000 | 0.977 |
| Average daily gain (g) | 381 | 375 | 378 | 370 | 12.02 | 0.000 | 0.972 |
| Average daily feed intake (g) | 563 | 558 | 597 | 569 | 14.33 | <0.001 | 0.233 |
| Feed conversion ratio | 1.49 | 1.50 | 1.61 | 1.61 | 0.04 | 0.527 | 0.523 |
| Faecal consistency score | 0.31 | 0.31 | 0.37 | 0.38 | 0.05 | 0.028 | 0.908 |

SEM – standard error of the mean
