# Supplementary material for: Dietary Ulva lactuca and CAZyme supplementation improve serum biochemical profile and hepatic composition of weaned piglets
Source: Sci Rep. 2023 May 31;13:8784. doi: 10.1038/s41598-023-36008-4 (PMC10232413; doi:10.1038/s41598-023-36008-4)
Supplement: Supplementary file 2 — Supplementary Table 2. [file 41598_2023_36008_MOESM2_ESM.docx]

**Supplementary material 2 – Plot loadings of Principal Components Analysis**

Table 1 – Plot loadings of Principal Components Analysis of serum metabolites.

|  | Dim.1 | Dim.2 |
| --- | --- | --- |
| Cholesterol | -0.845 | 0.433 |
| Urea | -0.811 | -0.209 |
| Total lipids | -0.787 | 0.560 |
| LDL | -0.721 | 0.446 |
| GGT | -0.717 | -0.082 |
| IgG | -0.624 | -0.631 |
| HDL | -0.587 | 0.455 |
| GPx | -0.091 | 0.149 |
| Triacylglycerols | -0.057 | 0.715 |
| VLDL | -0.057 | 0.715 |
| IGF1 | 0.038 | 0.857 |
| IgM | 0.043 | 0.130 |
| White blood cells | 0.097 | -0.269 |
| Granulocytes | 0.110 | -0.223 |
| Linfocytes | 0.110 | -0.168 |
| Monocytes | 0.133 | -0.190 |
| TAC | 0.158 | 0.465 |
| ALP | 0.214 | 0.738 |
| HOMAIR | 0.225 | 0.038 |
| Cl­ | 0.247 | -0.380 |
| IL10 | 0.268 | -0.232 |
| Total protein | 0.327 | 0.317 |
| Cortisol | 0.403 | 0.536 |
| Na^+^ | 0.419 | 0.110 |
| ALT | 0.427 | -0.034 |
| Albumin | 0.466 | 0.403 |
| Glucose | 0.470 | 0.388 |
| Creatinine | 0.473 | 0.499 |
| K^+^ | 0.580 | -0.392 |
| AST | 0.714 | -0.024 |

Table 2 – Plot loadings of Principal Components Analysis of hepatic metabolites.

|  | Dim.1 | Dim.2 |
| --- | --- | --- |
| SFA | -0.908 | -0.239 |
| C18 | -0.829 | 0.087 |
| C16 | -0.764 | 0.056 |
| C226*n-*3 | -0.672 | 0.373 |
| C205*n-*3 | -0.663 | -0.193 |
| C221*n-*9 | -0.620 | -0.468 |
| C181*c*11 | -0.580 | 0.228 |
| C161*c*7 | -0.517 | -0.019 |
| C20 | -0.495 | -0.634 |
| *n-*3PUFA | -0.477 | 0.641 |
| C22 | -0.393 | -0.635 |
| C181*c*9 | -0.324 | 0.259 |
| Ca | -0.311 | 0.119 |
| S | -0.311 | 0.696 |
| Mg | -0.245 | 0.207 |
| *Cis* MUFA | -0.170 | 0.168 |
| Fe | -0.156 | 0.386 |
| K | -0.123 | 0.641 |
| Zn | -0.118 | 0.300 |
| Total microminerals | -0.113 | 0.123 |
| C12 | -0.108 | -0.298 |
| Total macrominerals | -0.049 | 0.669 |
| Total minerals | -0.047 | -0.058 |
| C14 | -0.039 | -0.355 |
| Mn | 0.037 | -0.189 |
| C161*c*9 | 0.061 | 0.240 |
| C183*n-*6 | 0.115 | -0.305 |
| Na | 0.116 | -0.051 |
| P | 0.134 | 0.401 |
| C225*n-*3 | 0.142 | 0.666 |
| C201*c*11 | 0.194 | -0.392 |
| C183*n-*3 | 0.251 | 0.625 |
| C184*n-*3 | 0.294 | 0.480 |
| Cu | 0.340 | 0.094 |
| Other | 0.447 | -0.169 |
| C17 | 0.638 | -0.578 |
| C202*n*-6 | 0.646 | -0.391 |
| C15 | 0.650 | -0.630 |
| C182*t*9*t*12 | 0.655 | 0.372 |
| C171*c*9 | 0.731 | -0.342 |
| C203*n-*6 | 0.832 | 0.272 |
| C204*n-*6 | 0.924 | 0.246 |
| C182*n-*6 | 0.927 | 0.132 |
| PUFA | 0.934 | 0.205 |
| *n-*6 PUFA | 0.944 | 0.171 |
